# Chromosome 1q gain and tenascin-C expression are candidate markers to define different risk groups in pediatric posterior fossa ependymoma

**DOI:** 10.1186/s40478-016-0349-9

**Published:** 2016-08-22

**Authors:** Asuka Araki, Monika Chocholous, Johannes Gojo, Christian Dorfer, Thomas Czech, Harald Heinzl, Karin Dieckmann, Inge M. Ambros, Peter F. Ambros, Irene Slavc, Christine Haberler

**Affiliations:** 1 Institute of Neurology, Medical University of Vienna, Vienna, Austria; 2 Department of Pediatrics and Adolescent Medicine, Medical University of Vienna, Vienna, Austria; 3 Department of Neurosurgery, Medical University of Vienna, Vienna, Austria; 4 Center for Medical Statistics, Informatics, and Intelligent Systems, Medical University of Vienna, Vienna, Austria; 5 Department of Radiotherapy, Medical University of Vienna, Vienna, Austria; 6 Comprehensive Cancer Center, Medical University of Vienna, Währinger Gürtel 18-20, A-1097 Vienna, Austria; 7 Children’s Cancer Research Institute, Vienna, Austria; 8 Present address: Organ Pathology Unit, School of Medicine, Shimane University, Izumo, Japan

**Keywords:** Ependymoma, Pediatric, Prognostic markers, Chromosome 1q, Tenascin-C

## Abstract

Intracranial classic (WHO grade II) and anaplastic (WHO grade III) ependymomas are among the most common tumors in pediatric patients and have due to frequent recurrences and late relapses a relatively poor outcome. The impact of histopathological grading on patient outcome is controversial and therefore, molecular prognostic and predictive markers are needed to improve patient outcome. To date, the most promising candidate marker is chromosome 1q gain, which has been associated in independent studies with adverse outcome. Furthermore, gene expression and methylation profiles revealed distinct molecular subgroups in the supratentorial and posterior fossa (PF) compartment and Laminin alpha-2 (LAMA2) and Neural Epidermal Growth Factor Like-2 (NELL2) were suggested as surrogate markers for the two PF subgroups PF-EPN-A and PF-EPN-B. PF-EPN-A tumors were also characterized by tenascin-C (TNC) expression and *tenascin-C* has been suggested as candidate gene on 9q, involved in tumor progression. Therefore, we have analyzed the status of chromosome 1q, TNC, LAMA2, and NELL2 expression in a series of pediatric PF ependymomas in terms of their frequency, associations among themselves, and clinical parameters, as well as their prognostic impact. We confirm the negative prognostic impact of 1q gain and TNC expression and could classify PF ependymomas by these two markers into three molecular subgroups. Tumors with combined 1q gain and TNC expression had the poorest, tumors without 1q gain and TNC expression had a favorable and TNC positive 1q non-gained cases had an intermediate outcome. We found also differences in age and tumor grade in the three subgroups and thus, provide evidence that PF pediatric ependymomas can be divided by chromosome 1q status and TNC expression in three molecular subgroups with distinct clinico-pathological features. These analyses require only few amounts of tumor tissue, are broadly available in the routine clinical neuropathological setting and thus, could be used in further therapy trials to optimize treatment of ependymoma patients.

## Introduction

Ependymomas are neuroepithelial tumors, which arise throughout the neuraxis and are usually associated with the ventricular cavities or the spinal central canal. They are considered to originate from radial glial stem cells [1] and may occur at any age. Yet, distribution of localization, genetic alterations, and outcome differ between adults and children [2], necessitating different considerations regarding biology and treatment approaches for the different age groups. In the pediatric population ependymomas represent the fourth most common central nervous system (CNS) tumor after low- and high-grade gliomas and medulloblastomas [3]. In patients below 18 years, they account for 5.5 % of all CNS tumors and this frequency increases to 10.4 % in patients below 14 years with a peak incidence in children between 1 and 4 years [3]. In contrast to adults where spinal localization predominates, ependymomas in children are located predominantly intracranially, and most commonly in the posterior fossa [2]. Outcome of pediatric patients with intracranial ependymoma remains relatively poor with 5-year progression free survival rates between 30 % and 69.1 % and 5 year overall survival rates between 60 and 81 % in different age groups and therapeutic trial cohorts [4–7]. Among clinical factors the extent of resection is the most important prognostic factor for patient outcome [8], but age below 3 years and infratentorial location have been also suggested as unfavorable prognostic markers [9, 10]. Histopathologically, intracranial ependymomas comprise subependymoma (WHO grade I), which is generally associated with a favourable biological behavior, as well as classic (WHO grade II) and anaplastic ependymomas (WHO grade III) [11]. Data on the prognostic impact of tumor grade i.e. classic versus anaplastic on patient outcome are controversial. Whereas in some studies a better outcome of patients with classic ependymomas was found [10, 12–14], this could not be confirmed in other studies [15]. Thus, the biological behavior of ependymomas WHO grade II and III is still poorly predictable, and there is a need for robust molecular prognostic and predictive markers to optimize treatment strategies and improve patient outcome.

In the past years numerous markers including Ki67 proliferation, EGFR expression, Nestin, EZH2, human telomerase reverse transcriptase hTERT/NCL, gain of chromosome 9q, tenascin-C (TNC) and chromosome 1q gain have been associated with patient outcome [16–21] for review see [22]. Among these markers chromosome 1q gain seems to be the most promising marker, as its negative prognostic impact could be shown in several studies [17, 23–26]. Furthermore, recently two distinct molecular ependymoma subgroups could be defined in the infra- and supratentorial compartment, respectively [27, 28]. The two supratentorial subgroups are characterized by fusions of the *RELA* or *YAP1* gene, respectively [27, 29, 30] and patients with *RELA* fusion were shown to have a worse prognosis in one study [27]. Posterior fossa tumors could be classified into Group A (EPN-PF-A) and B (EPN-PF-B) tumors [27, 28]. Group A patients are younger, have frequently tumors with a balanced genome and a worse prognosis compared to Group B patients. Immunohistochemical expression of LAMA2 and NELL2 were proposed as markers for EPN-PFA and EPN-PFB tumors, respectively and high tenascin-C (TNC) expression was described in EPN-PFA tumors [28].

However, to date none of these markers is used for therapy stratification. Therefore, the aim of this study was to analyze in an ependymoma series, including children and adolescents younger than 21 years, candidate molecular markers, which require only few amounts of tumors tissue and can be easily performed in a routine clinical neuropathology setting and thus, might be used in upcoming clinical therapeutic trials for therapy stratification.

## Materials and methods

### Patients

In the present study 52 children and adolescents (≤21 years) operated on and/or treated for an intracranial posterior fossa (PF) ependymoma at the Medical University of Vienna between 1965 and 2014 were included. Patients with subependymoma were excluded. Clinical characteristics of the patients including treatment are presented in Table 1.

**Table 1:** Clinical and biological characteristics of 52 pediatric posterior fossa (PF) ependymomas

Variables	*n =* 52 (%)
Age (years)	
range	0.9–20.4
median	3.1
Gender	
female	24 (46.2)
male	28 (53.8)
Extent of resection	
GTR	34 (65.4)
non-GTR	17 (32.7)
n.a	1 (1.9)
Adjuvant therapy	
no	9 (17.3)
RTX	14 (26.9)
CTX	5 (9.6)
RTX and CTX	24 (46.1)
WHO grade	
grade II	18 (34.6)
grade III	34 (65.4)
TNC	
positive	46 (88.5)
negative	6 (11.5)
LAMA2	
positive	28 (53.8)
negative	24 (46.2)
Chromosome 1q	
gain	10 (19.2)
no gain	34 (65.4)
n.a.	8 (15.4)

Extent of tumor resection was determined by postoperative MRI within 72 hours of surgery from the early 1990s on. In earlier years the extent of tumor resection was judged by the neurosurgeon only and confirmed by CT when available and deemed necessary during the 1980s. This study was approved by the Ethics Committee of the Medical University of Vienna.

### Histopathology

To confirm the diagnosis of ependymoma, tumor tissue of all patients was reviewed without knowledge of clinical information by A.A and C.H. Routine immunohistochemical stainings with antibodies against Olig2, Vimentin, GFAP, EMA, NFP, NeuN, Synaptophysin, CD34, and Ki67 were performed in all tumors. In 17 anaplastic tumors with structures reminiscent of multilayered rosettes, anti-LIN28A immunostaining (clone A177, Cell Signaling, Danvers, USA; dilution 1:100) was conducted to exclude an embryonal tumor with multilayered rosettes (ETMR). In 2 of the 52 patients only tissue of the local relapse was available for analyses. In 4 patients tissue of the primary and the recurrent tumor (3 local, 1 metastatic) was analysed. Histopathological grading was performed according to the criteria of the WHO classification of central nervous system tumors and recent studies based on cellularity, frequency of mitotic figures, and microvascular proliferations [11, 14, 24].

### Immunohistochemistry

For immunohistochemical analyses 3 μm thick sections of FFPE tumor tissue were cut. Antibodies against TNC (clone E-9, Santa Cruz, Dallas, USA, dilution, 1:150), LAMA2 (clone 2D4, Abnova, Taipei, Taiwan, dilution 1:500) and NELL2 (Abcam, Cambridge, United Kingdom, dilution 1:5) were used. The protocol consisted of antigen retrieval in a water bath at 98 °C using citrate buffer (pH 9.0, 20 min for TNC and NELL2; pH 6.0, 20 min for LAMA2). Detection of immunostaining was performed using the Envision rabbit/mouse detection system (K5007, Dako, Glostrup, Denmark) and diaminobenzidine as chromogen. Lung squamous cell carcinoma, normal adult pancreas and hippocampus were used as positive controls for TNC, LAMA2 and NELL2, respectively. Despite different pretreatment protocols and antibody dilutions, no reliable and reproducible results could be obtained for the NELL2 antibody. Therefore, we refrained from evaluation of the staining. According to previous studies the expression of TNC and LAMA2 was scored as positive or negative [18, 28, 31], labeling of vessel walls served as internal positive control. TNC immunoreactivity was extracellular and different expression patterns including predominantly perivascular areas, the central or the border-zone of the tumor tissue, as well as a mixture of these patterns was observed. LAMA2 expression was cytoplasmic.

### Interphase fluorescent in situ hybridization (FISH)

Dual color FISH was carried out on FFPE sections as previously described [32] using a commercially available 1q25 (spectrum green) and 1p36 (spectrum orange) probe (Vysis® Abbott Laboratories, Abbott Park, IL, USA). FISH slides were examined with an AxioImager Z1 microscope (Carl Zeiss Microscopy, New York, USA) using the ISIS software from MetaSystems (Altlussheim, Germany). Signals were counted with x63 magnification in at least 200 non-overlapping tumor cell nuclei in each section. A signal ratio of 1q25/1p36 > 1 in a single nucleus was considered as gain.

### SNP arrays

In three patients SNP arrays were performed as previously published [33] to confirm the results of the FISH analyses.

### Statistical analyses

For statistical analysis, SPSS version 22.0 (SPSS Inc., Chicago, Illinois, USA) and SAS version 9.4 (SAS Institute Inc., Cary, NC, USA) were used. Associations between biological and clinical markers were assessed with Spearman’s rank correlation coefficient. Overall survival (OS) was defined from date of first operation until date of death. Progression free survival (PFS) was defined from date of first operation until date of disease progression, disease recurrence or death. Survival times of patients still alive or progression free at the end of the observation period were considered censored with the date of last contact. Survival probabilities were calculated with the product limit method of Kaplan and Meier and corresponding confidence limits were calculated by applying the log-log transformation. The log-rank test as well as univariate and multivariable Cox proportional hazards regression models were used to assess the effects of variables of interest on OS and PFS. Whenever it was necessary to address numerical problems in the Cox model due to small sample sizes, the Firth-correction was applied and Profile Likelihood Confidence Limits were used [34]. All p-values are results of two-sided tests; values of *p <* 0.05 were considered statistically significant. As this study is exploratory rather than confirmatory no adjustments for multiple testing have been performed.

## Results

### Tumor grade, immunohistochemical and FISH analyses

**Fig. 1:**
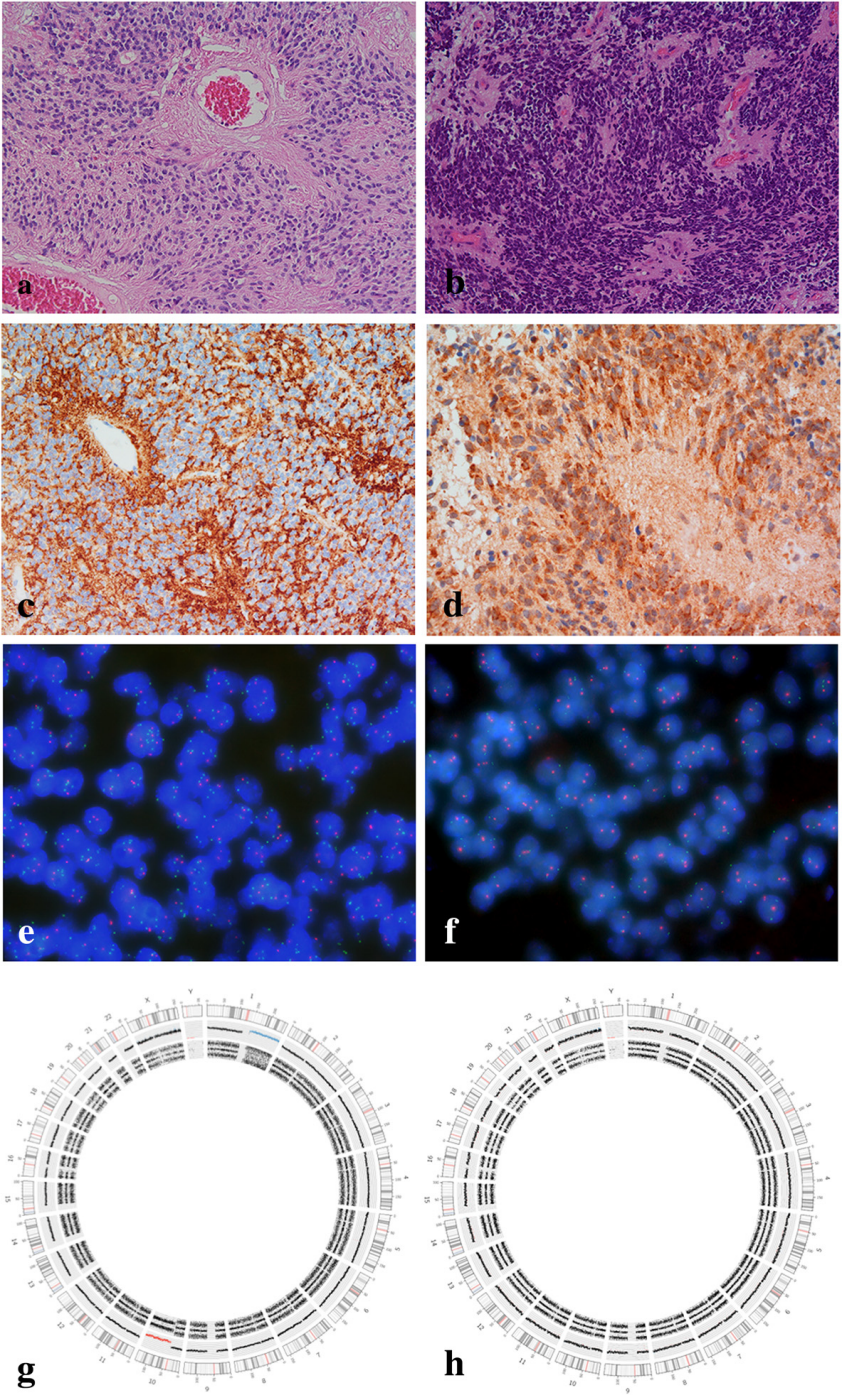
Histopathology and molecular markers in ependymoma. Classic expendymoma WHO grade II (**a**); anaplastic ependymoma WHO grade III (**b**); ependymoma with TNC (**c**) and LAMA2 (**d**) expression; ependymoma with (**e**) and without (**f**) 1q25 gain (1q25 green fluorochrome, 1p36 red fluorochrome); g, h confirmation of iFSIH results by SNP arrays, Circos plots showing gain of chromosome arm 1q (**g**) and a balanced copy-number profile (**h**)

Tumor characteristics including tumor grade and results of immunohistochemical analyses are summarized in Table 1. Briefly, 34.6 % of the tumors were classified as grade II (Fig. 1a) and 65.4 % as grade III (Fig. 1b). TNC immunoreactivity was extracellular (Fig. 1c) and different expression patterns including predominantly perivascular areas, the central or the border-zone of the tumor tissue, as well as a mixture of these patterns was observed. LAMA2 expression was cytoplasmic (Fig. 1d). TNC and Lama2 expression was detectable in 88.5 % and 53.8 % of the tumors, respectively. In 4 patients, we analysed TNC expression in both, the primary and recurrent tumor and found an identical pattern (all positive).

FISH analysis revealed chromosome 1q25 gain (Fig. 1e) in 10 tumors, 34 tumors had a balanced chromosome 1 (Fig. 1f). In 8 tumors (all operation dates before 1992) no FISH signals were detectable despite repeated analyses. The percentage of tumor cell nuclei with chromosome 1q25 gain (signal ratio 1q25/1p36 > 1 in a single cell) ranged from 40.8 % to 83.5 % (median: 54.5 %) in gained and from 0 % to 6.5 % (median 2 %) in balanced cases. To corroborate the FISH results, we performed SNP arrays in 2 tumors with chromosome 1q25 gain (Fig. 1g) and in 1 tumor with balanced chromosome 1 (Fig. 1h). The FISH results could be confirmed in all three cases. All three tumors with 1q25 gain had a gain of the whole chromosome arm 1q. Analysis of chromosome 1q25 in matched recurrences revealed in three tumors the same result as in the primary lesion (2 cases with 1q25 gain, 1 balanced case). In one local recurrence *de novo* 1q25 gain was detected.

### Associations between clinical, pathological and molecular markers

The results of association analyses between clinical, pathological and molecular markers are provided in Table 2. No significant associations were detected for gender and extent of resection (data not shown). TNC and LAMA2 expression were significantly associated with younger patient age. TNC positive tumors were more frequently anaplastic ependymomas, whereas this association could not be found for LAMA2 positive tumors. All tumors with chromosome 1q25 gain were anaplastic and TNC positive.

**Table 2:** Association of clinical and biological markers in PF ependymomas, Spearman correlation coefficients

Markers	Age	WHO grade	TNC	LAMA2
Age				
WHO grade	–.263			
II/III				
TNC	–.452**	.496**		
negative/positive				
LAMA2	–.308*	.056	.149	
negative/positive				
1q25 gain	.265	.351*	.194	–.158
absent/present				

### Survival analyses

At the last follow-up 29 (55.8 %) patients were alive. Six patients were excluded from survival analyses because five died perioperatively (operation dates before 1990), and one died of another cause. The 5-year and 10-year survival was 51.6 % (95 % confidence interval (CI) = 35.8–5.3) and 46 %. (95 % CI = 30.4–60.3). Median follow-up was 174.7 months (range: 8.5–452.3 months). Yet, before 1992 not all patients were treated with irradiation, whereas after 1992, all patients primarily managed at the Medical University of Vienna—independent of age—were irradiated at least focally and received chemotherapy. Consequently, we found significant differences in overall survival (OS) between patients operated before and after 1992 (5-and 10- year OS 31.6 % (95 % CI = 12.9–52.2 %) and 26.3 % (95 % CI =9.6–46.8) versus 65.6 % (95 % CI =42.5–81.3) and 60.6 %, (95 % CI =37.5–77.5) *p <* 0.001). Therefore, survival analyses were primarily calculated for patients operated after 1992 (recent cohort) and in a second step for all patients (entire cohort). Progression free survival (PFS) data were available only for patients operated after 1992. 5-year and 10-year PFS were 56.7 % (95 % CI =35.2–73.4) and 51.1 % (95 % CI =29.4–69.1), respectively.

**Fig. 2:**
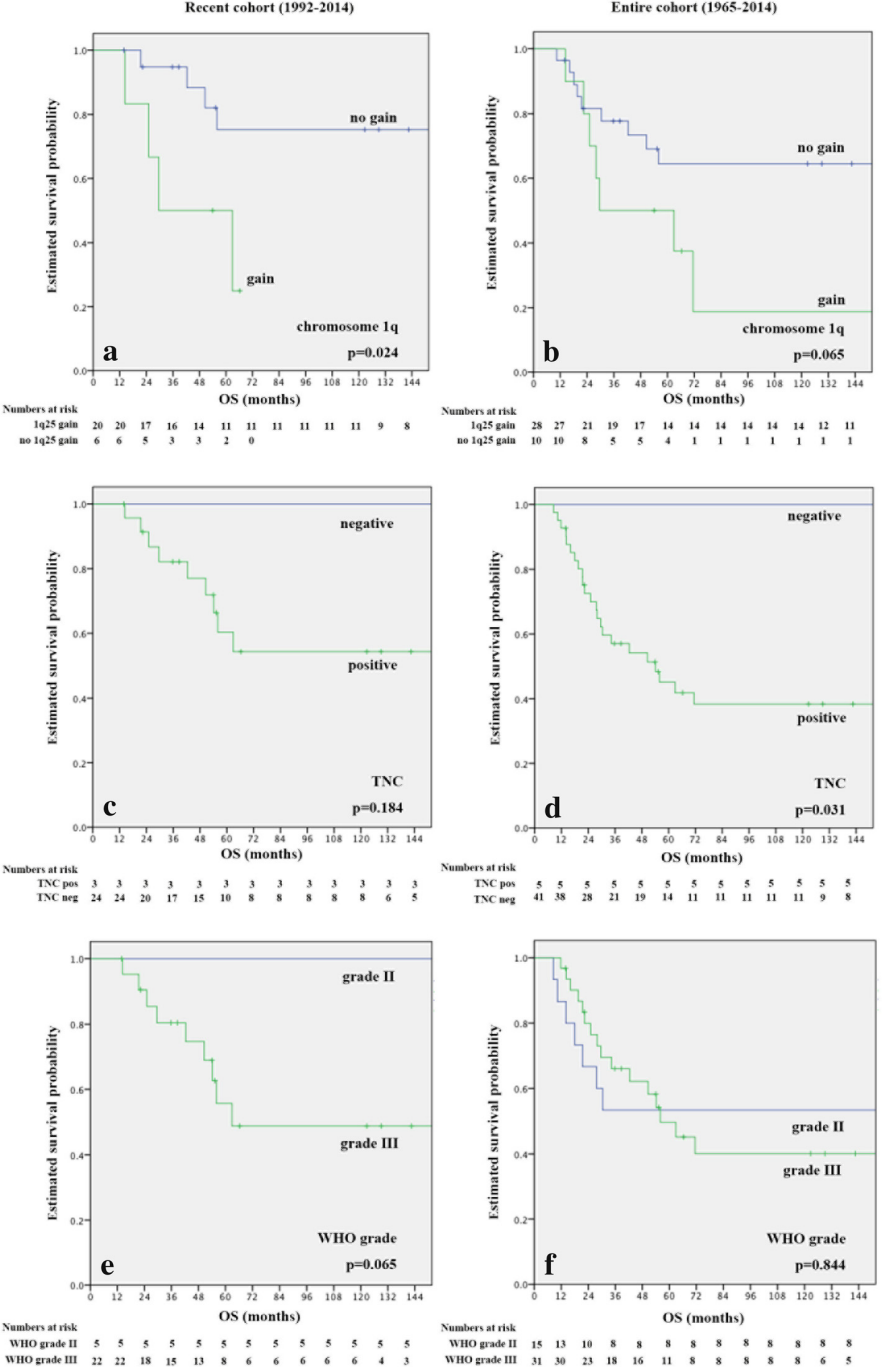
Kaplan Meier overall survival curves for chromosome 1q status (**a**, **b**), TNC (**c**, **d**) and WHO grade (**e**, **f**) in the recent (1992–2014; **a**, **c**, **e**) and the entire cohort (1965–2014; **b**, **d**, **f**)

Results of survival analyses in the recent (1992–2014) and the entire cohort (1965–2014) are presented in Table 3. In brief, presence of chromosome 1q25 gain had a significant negative impact on survival in the recent (Fig. 2a) and approached significance in the entire cohort (Fig. 2b). Presence of TNC expression did not show a significant effect on OS, but approached significance for PFS in the recent cohort (Fig. 2c), and had a significant negative impact on OS in the entire cohort (Fig. 2d). LAMA2 expression was not significantly associated with survival. WHO grade nearly reached significance in the recent cohort (Fig. 2e), but was not significant in the entire cohort (Fig. 2f). Extent of resection did not significantly influence OS in the recent cohort, probably because only 3 patients did not have gross total resection, but had a significant impact on PFS as well as on OS in the entire cohort. No significant influence of age and localization was detectable (data not shown).

**Table 3:** Survival analyses of clinical and biological markers in the recent and the entire cohort

Parameters	Overall survival		Progression free survival		Overall survival	
	1992–2014		1992–2014		1965–2014	
	(*n =* 27)		(*n =* 27)		(*n =* 46)	
	HR (95 % CI)	*p*-value	HR (95 % CI)	*p*-value	HR (95 % CI)	*p*-value
*Univariate*						
Age (years)	0.957 (0.807; 1.135)	0.609	0.930 (0.800; 1.080)	0.324	0.956 (0.875; 1.046)	0.322
Extent of resection GTR/non-GTR	1.416 (0.173; 11.57)	0.744	5.379 (1.068; 27.08)	**0.042**	2.876 (1.243; 6.657)	**0.010**
WHO grade II/III	7.289 (0.911; 942.8)	0.065	2.421 (0.516; 11.36)	0.249	1.094 (0.448; 2.670)	0.844
TNC positive/negative	3.739 (0.470; 483.4)	0.184	6.624 (0.841; 856.9)	0.079	9.583 (1.323; 1219)	**0.031**
LAMA2 positive/negative	1.800 (0.369; 8.776)	0.461	1.701 (0.466; 6.209)	0.416	1.141 (0.497; 2.619)	0.756
Chromosome 1q25 gain/no gain	4.374 (1.085; 17.63)	**0.024**	4.855 (1.342; 17.56)	**0.008**	2.474 (0.915; 6.692)	0.065
*Multivariable* (*n =* 26/26/38)						
TNC positive/negative	1.872 (0.067; 52.31)	0.712	4.593 (0.505; 608.6)	0.348	3.807 (0.453; 496.7)	0.391
Chromosome 1q25 gain/no gain	4.409 (0.894; 21.74)	0.068	5.204 (1.369; 21.76)	**0.024**	2.025 (0.745; 5.307)	0.171
Extent of resection GTR/non-GTR	2.662 (0.767; 9.235)	0.123	2.585 (0.908; 6.235)	0.057	2.135 (0.933; 4.464)	0.062

Hazard ratios (HR), 95 % confidence intervals (CI) and *p*-values are presented

**Fig. 3:**
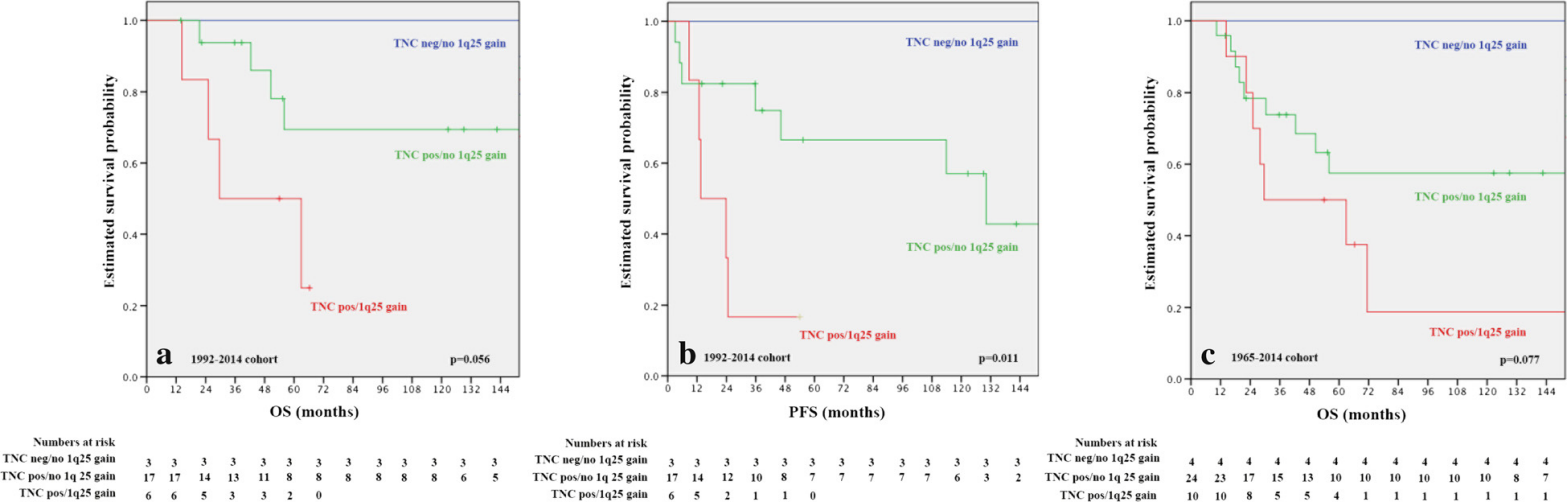
Survival probabilities for three molecular subgroups: OS (**a**) and PFS (**b**) in the recent cohort (1992–2014) and (**c**) OS in the entire cohort (1965–2014)

In multivariable analyses including chromosome 1q25 status and TNC expression, only gain of chromosome 1q25 retained an independent prognostic marker for PFS (Table 3). However, in view of the finding that TNC expression had a significant impact in the entire cohort, we defined three ependymoma groups using chromosome 1q25 status and TNC expression. We found significant differences in PFS (Fig. 3b) and significance was closely approached also in OS analyses (Fig. 3a, c). Patients harboring combined 1q25 gain and TNC expression had the poorest prognosis, followed by TNC positive/1q25 non-gained tumors, whereas patients with TNC negative/1q25 non-gained tumors had the best outcome. We further analyzed clinical characteristics and distribution of tumor grade in the three molecular subgroups, in the entire cohort (1965–2014) (Table 4). The median age of patients with TNC positive/1q25 non-gained tumors (group 2) was younger (2.14 years) than in those with TNC positive/1q25 gained tumors (group1) (4.5 years) and TNC negative/1q25 non-gained tumors (group 3) (9.9 years). 4 of 5 patients with TNC negative/1q25 non-gained tumors were male and had a classic ependymoma (WHO grade II). All PF TNC positive/1q25 gained tumors were anaplastic (grade III), and all TNC negative/1q25 non-gained tumors were classic (grade II) ependymomas, whereas TNC positive/1q25 non-gained tumors comprised classic (*n =* 8) and anaplastic (*n =* 21) ependymomas. In this subgroup no impact of tumor grade on survival was detectable (*p >* 0.05).

**Table 4:** Clinical characteristics and distribution of WHO grade in three different ependymoma groups defined by TNC expression and chromosome 1q25 status

	Gender (m:f ratio)	Median age years (range)	WHO grade (II/III)
TNC pos/1q25 gain (group 1)	1:1	4.2 (1.4–17.3)	0/10
TNC pos/no 1q25 gain (group 2)	1.4:1	2.1 (0.9–11.3)	8/21
TNC neg/no 1q25 gain (group 3)	4:1	9.9 (3.6–20)	5/0

## Discussion

The aim of this study was to analyze the status of chromosome 1q, TNC, LAMA2, and NELL2 expression in a series of pediatric PF ependymomas in terms of their frequency, associations among themselves and clinical parameters, as well as their prognostic impact.

In intracranial ependymomas gain of chromosome 1q is a frequent genomic aberration. It could be detected in up to 24 % of all pediatric cases [13, 17, 23–26], whereas it was found only in 8 % of adult patients in a metanalysis [22]. In line with these previous findings we detected 1q gain in our series in 19.2 %. As previously described gain of chromosome 1q was in our series associated with anaplastic tumors (WHO grade III) [1, 17, 23, 26, 35–38]. In 2012, two studies confirmed the negative prognostic impact of 1q gain on survival in pediatric patients treated within the frame of therapeutic trials [24, 25]. Kilday et al. described 1q gain as an independent prognostic marker for PFS but not OS in three clinical trial cohorts including ST and PF tumors. Godfraind et al. found a negative prognostic impact on OS and PFS in uni- and multivariable analyses in PF tumors. A recent study delineating molecular subgroups in the spinal, PF and ST compartment in a mixed pediatric and adult population confirmed 1q as an independent prognostic factor [27]. No prognostic impact of 1q gain was found only in 2 studies [13, 37], one of them could be due to low statistical power, because only 6 tumors with 1q gain were included [13]. We can confirm in our retrospective study in an independent series of intracranial ependymomas including children and adolescents below 22 years the adverse prognostic impact of 1q gain on OS and PFS, thus providing further evidence for the prognostic significance of chromosome 1q status. Interestingly, of the three patients with 1q gain still alive, one female patient is surviving 23 years after diagnosis. She was 4.4 years at diagnosis, had a gross total resection and received an intense therapy including RTX and CTX. This case highlights the possibility that even patients with adverse molecular prognostic markers may be cured. This is in line with a previous study describing 1q25 gain in the CNS9904/RT group treated by radiotherapy as less predictive of a worse patient PFS or OS than in chemotherapy trial cohorts [25]. The authors of this study suggested that primary radiotherapy is an effective counteractive adjuvant measure despite the adverse effects of 1q25 gain.

In our series of 4 matched primary and recurrent tumors 1 case showed a *de novo* 1q25 gain in the recurrent tumor. This is in accordance with previous data showing *de novo* 1q gain in single recurrences [23, 35, 39]. The patient in our series with *de novo* 1q gain in a locally recurrent tumor, which occurred 10 years after the primary tumor, had initially a TNC positive grade II ependymoma, but is now after reoperation and 3 blocks of PEI chemotherapy (VP16, Ifosfamid, Cisplatin), intraventricular VP16 and DepoCyte, and focal radiotherapy in complete remission 69 months after operation of the relapse. This case emphasizes the occurrence of very late relapses in ependymomas and thus, the importance of long-term follow-up, as well as the possibility to rescue patients with recurrence, even if their tumor harbors biologically unfavourable molecular features.

The extracellular matrix glycoprotein TNC is in the CNS widely expressed during early development in zones of proliferation, migration and morphogenesis [40]. During further differentiation TNC is progressively down-regulated, and in the adult CNS TNC expression remains adjacent to areas of active neurogenesis such as the hippocampus and the borders of the subventricular zone (see refs [40, 41] and references therein). However, it is actively re-expressed in the adult brain under pathological conditions such as infection, inflammation, wound healing or tumorigenesis [40]. It could be shown that gliomas grade II and III with pronounced perivascular TNC staining had a shorter disease- free time and in vitro TNC blocking antibodies inhibited proliferation and migration in glioblastoma [42]. Furthermore, targeting TNC by radiolabeled antibodies and RNAi have shown promising results in patients with malignant gliomas (for review see [43]). In ependymomas TNC expression has been associated with anaplastic tumors [31, 44–46]. A significant impact of TNC expression on PFS could be shown in two studies [31, 46]. *TNC* has been described as candidate gene for ependymoma progression in posterior fossa tumors with gain of 9pter [18]. TNC expression was found in 94 % of posterior fossa Group A tumors and only in 11 % of posterior fossa Group B tumors [28] and thus, seems to be a marker for the majority of PF Group A ependymomas. We found TNC expression in almost 90 % of the tumors. In line with previous reports it was significantly associated with anaplastic tumors [31, 44–46]. Similar to the report by Modena we found an association with younger patient age [47]. We detected a significant impact of TNC expression on OS in our entire cohort (1965–2014), and a tendency towards significance on PFS in the recent cohort. A possible explanation for the lack of significance in the recent cohort could be that the biological impact of TNC is blurred by improved adjuvant treatment.

LAMA2 (Laminin alpha-2) expression has been suggested as a surrogate marker for PF Group A ependymomas [28]. We found LAMA2 expression in approximately 50 % of all PF tumors. In a previously analyzed cohort of PF ependymomas LAMA2 was expressed in 67 % of the tumors in patients ≤ 21 years, thus in a similar percentage, however 10 % of these patients expressed also NELL2 [28]. In our series, no significant association between LAMA2 and TNC was detectable and we could not find a prognostic impact of LAMA2. NELL2 (Neural Epidermal Growth Factor Like-2), which has been suggested as surrogate marker for PF Group B ependymomas [28], did not show reproducible results in our hands, thus this marker does not seem to be a useful, robust marker for routine clinical use.

As we detected a prognostic impact of chromosome 1q status and TNC expression we combined these two markers and defined 3 molecular subgroups. Actually, we found survival differences in OS and PFS. Patients whose tumors harbored combined 1q gain and TNC expression (group 1) had the poorest prognosis, whereas no 1q gain and lack of TNC expression (group 3) was associated with a favorable outcome. Group 1 and 2 probably correspond to the previously described PF-EPN-A and group 3 to the PF-EPN-B subgroup [27, 28]. Group 3 tumors might represent a very favorable molecular group and patients with such tumors might be treated less aggressively, thereby reducing long-term side effects. Indeed, one 11-year-old male patient with a tumor without 1q25 gain and TNC expression, who had a gross total resection did not receive any adjuvant treatment and is alive 35 years after operation. We further analyzed whether these three molecular subgroups are associated with distinct clinico-pathological features (Table 4). Actually, the median age of patients with group 2 tumors was younger than that of patients with group 1 tumors, and median age of patients with group 3 tumors was highest. There were also differences regarding the tumor grade, whereas all group 1 tumors were anaplastic (WHO grade III), all group 3 tumors were classic (WHO grade II) and we found a preponderance of male patients in group 3 tumors. This further provides evidence that the three groups represent biologically distinct subgroups, and that PF-EPN-A tumors can be split into 2 subgroups with different outcome and clinico-pathological features.

## Conclusions

In summary, we confirm in an independent series of pediatric ependymoma patients the negative prognostic impact of chromosome 1q25 gain and TNC expression in PF ependymomas and provide evidence that PF ependymomas can be split into three different molecular subgroups with distinct clinico-pathological features and outcome, which could be used in future trials to optimize treatment of ependymoma patients.
